# Inflammatory cytokine profile and T cell responses in African tick bite fever patients

**DOI:** 10.1007/s00430-022-00738-5

**Published:** 2022-05-11

**Authors:** Jessica Rauch, Johannes Jochum, Philip Eisermann, Jana Gisbrecht, Katrin Völker, Friederike Hunstig, Ute Mehlhoop, Birgit Muntau, Dennis Tappe

**Affiliations:** 1 Bernhard Nocht Institute for Tropical Medicine, Bernhard-Nocht-Str. 74, 20359 Hamburg, Germany; 2 University Medical Center Hamburg-Eppendorf, Hamburg, Germany; 3 Bundeswehrkrankenhaus, Hamburg, Germany

**Keywords:** *Rickettsia africae*, African tick bite fever, Cytokines, Interleukin 22, Interferon, T cells

## Abstract

African tick bite fever, an acute febrile illness, is caused by the obligate intracellular bacterium *Rickettsia africae*. Immune responses to rickettsial infections have so far mainly been investigated in vitro with infected endothelial cells as the main target cells, and in mouse models. Patient studies are rare and little is known about the immunology of human infections. In this study, inflammatory mediators and T cell responses were examined in samples from 13 patients with polymerase chain reaction-confirmed *R. africae* infections at different time points of illness. The Th1-associated cytokines IFNγ and IL-12 were increased in the acute phase of illness, as were levels of the T cell chemoattractant cytokine CXCL-10. In addition, the anti-inflammatory cytokine IL-10 and also IL-22 were elevated. IL-22 but not IFNγ was increasingly produced by CD4^+^ and CD8^+^ T cells during illness. Besides IFNγ, IL-22 appears to play a protective role in rickettsial infections.

**Supplementary Information:**

The online version contains supplementary material available at 10.1007/s00430-022-00738-5.

## Introduction

African tick bite fever (ATBF), a spotted fever group (SFG) rickettsiosis, is an acute febrile illness seen particularly in rural sub-Saharan Africa. Increasing travel activities to tropical and sub-tropical regions, especially safari tourism, has led to an increased incidence of ATBF among returning travelers [1–4]. The causative agent of ATBF is *Rickettsia africae*, an obligate intracellular Gram-negative bacterium, which is transmitted to humans by ticks of the *Amblyomma* genus, more precisely *A. hebraeum* and *A. variegatum* [5]. *Amblyomma* ticks are rather aggressive and may bite the host several times leading to the development of multiple eschars at the site of bacterial inoculation in up to 50% of patients [4]. Flu-like symptoms such as fever, nausea, headache, fatigue and myalgia can occur in ATBF and 15–56% of patients show a generalized cutaneous rash [6]. Complications in ATBF seem to be rare as there are no reports of life-threatening complications or published fatal cases, which is in contrast to other SFG rickettsioses, such as Rocky Mountain spotted fever (RMSF), or the typhus group (TG) rickettsioses.

Endothelial cells (ECs) are the primary cells infected by rickettsiae and, as a consequence, vascular inflammation and impaired vascular integrity occur [7]. ECs release a multitude of cytokines and chemokines in vitro that can attract and activate immune cells to the site of infection and thus trigger inflammatory responses [8]. Investigations of immune responses in vivo have so far mainly been studied in murine models of rickettsial infections. In such mouse models, T cells (especially CD8^+^ T cells but also CD4^+^ T cells) and their immune mediators have been shown to play a dominant role in protection against rickettsial infections (reviewed in [9]). In contrast, patient studies are rare and little is known about the inflammatory mediators and T cell responses in human rickettsioses.

To shed more light on the immune responses in human ATBF, serum cytokine, chemokine and growth factor levels were examined and inflammatory T cell responses were analyzed from patients diagnosed with ATBF at the National Reference Centre for Tropical Pathogens in Hamburg, Germany, during 2015–2020.

## Patients, materials and methods

### Patients and controls

Thirteen ATBF patients were included in the study. All individuals were diagnosed at the National Reference Centre for Tropical Pathogens in Hamburg, Germany, during 2015–2020 (Table 1). The patients were tourists who returned from South Africa (11 patients), Botswana and Malawi (one patient each). Eight patients were men, five were women and they ranged in age from 36 to 76 years (median age 49 years).

**Table 1:** Characteristics of 13 patients with molecularly confirmed ATBF, Germany, 2015–2020

Patient no	Age/sex	Year of diagnosis	Travel history	Signs and symptoms	Hospitalization
1	41/male	2015	South Africa	Arthralgia, eschar on left lower leg, fever, headache, inguinal lymphadenopathy	No
2	46/male	2017	South Africa	Apathy, eschar on right lower leg, fever, chills, inguinal lymphadenopathy	Yes
3	44/female	2017	South Africa	Right inguinal eschar and lymphadenopathy	Yes
4	43/male	2019	South Africa	Apathy, eschar on right upper arm, headache, axillary lymphadenopathy	No
5	75/female	2019	South Africa	Two eschars on left thigh, diarrhea	No
6	60/female	2019	South Africa	Arthralgia, multiple eschars on both thighs, fever, headache, inguinal lymphadenopathy	No
7	60/male	2019	South Africa	Apathy, eschar on right thorax with concomitant lymphangitis, headache	No
8	53/male	2019	South Africa	Eschar on right upper arm, fever, headache	No
9	36/female	2019	South Africa	Eschar on left thigh, fever, headache, cervical and inguinal lymphadenopathy	No
10	57/male	2019	Malawi	Eschar on thigh, fever	No
11	49/male	2019	South Africa	Apathy, myalgia, multiple eschars on both legs, cervical and inguinal lymphadenopathy, increased insulin demand for type 1 diabetes mellitus	No
12	76/female	2019	South Africa	Myalgia, arthralgia, eschar on thigh, fever, headache	No
13	48/male	2020	Botswana	Myalgia, fatigue, sweating, white eschar on right thigh, lymphangitis, inguinal lymphadenopathy	No

**Fig. 1:**
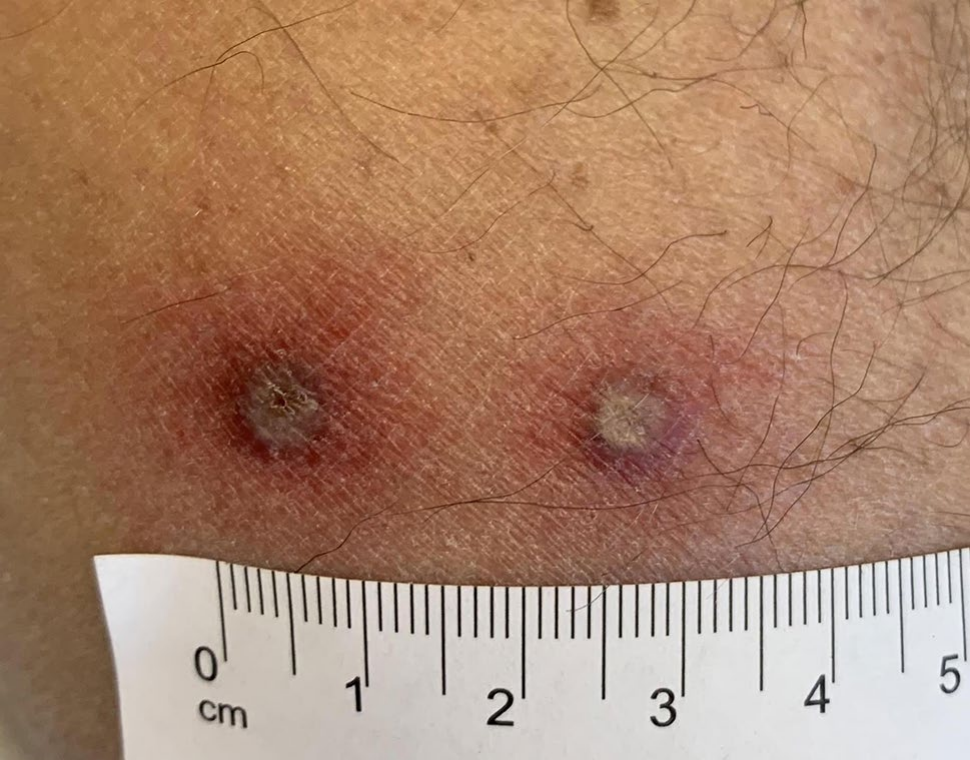
Cutaneous eschars from returning traveler with African tick bite fever. Two eschars in an early developmental stage are shown. For all patients in this study, African tick bite fever was diagnosed from eschar material based on positive real-time quantitative polymerase chain reaction testing and subsequent sequencing result for *R. africae*

In all patients, at least one eschar was found, multiple eschars (Fig. 1) were visible in 23% (3 patients) of the patients. Lymphadenopathy or lymphangitis was seen in 69% (9 patients), fever in 54% (7 patients), followed by headache in 54% (7 patients), followed by apathy or fatigue in 39%, (5 patients), and arthralgia and myalgia in 39% (5 patients) of the individuals. Two of the patients required hospitalization.

ATBF was diagnosed on the basis of a positive pan-rickettsial real-time quantitative polymerase chain reaction (qPCR) testing result targeting the bacterial *ompB* gene [10] from eschar material. A conventional gel PCR was used for later sequencing [11], followed by BLAST analysis (www.blast.ncbi.nlm.nih.gov) with positive results (99–100% sequence homology) for *R. africae* in all patients. In addition, an in-house immunofluorescence antibody test (IFAT) was positive in serum from all individuals, with the exception of one patient who did not seroconvert during the study period (patient 6; Table 2). One patient showed only IgG and no IgM in the IFAT during an early blood sampling date, possibly reflecting a previous infection (patient 10). IFAT was performed using *R. africae* strain ESF-5 grown in L929 mouse fibroblast cell culture. The IFAT cutoff values for *R. africae* were < 1:40 (IgM and IgG), the cutoff values were determined with sera from 44 healthy Caucasian blood donors who all had titers below the cutoff. Sera were used in a dilution of 1:40 and 1:80 for screening, employing secondary fluorescein-coupled antibody conjugates (Jackson ImmunoResearch, Ely, United Kingdom) in dilutions of 1:80 (IgM) and 1:200 (IgG). If positive, sera were further titrated in a serial dilution.

**Table 2:** Serologic testing results for 13 patients with African tick bite fever, Germany 2015–2020

Patient no	Day of illness	IgM against *R. africae*	IgG against *R. africae*	Cytokine measurements	Analysis of T cell responses
1	6	1:80	< 1:40	Yes (early acute)	n.d.
2	11	1:40	< 1:40	Yes (late acute)	Yes
	15	1:80	1:640	Yes (convalescent)	n.d.
3	10	1:40	< 1:40	Yes (late acute)	n.d.
	23	1:40	1:80	Yes (convalescent)	n.d.
4	11	1:80	1:40	Yes (late acute)	Yes
5	1	< 1:40	< 1:40	Yes (early acute)	Yes
	13	1:40	1:160	Yes (late acute)	Yes
6	2	< 1:40	< 1:40	Yes (early acute)	Yes
7	5	< 1:40	< 1:40	Yes (early acute)	Yes
	29	1:80	1:320	Yes (convalescent)	Yes
8	10	1:40	< 1:40	Yes (late acute)	Yes
	39	1:40	1:80	Yes (convalescent)	Yes
9	17	1:40	1:160	Yes (convalescent)	Yes
10	2	< 1:40	1:160	Yes (early acute)	n.d.
11	6	< 1:40	< 1:40	Yes (early acute)	n.d.
	37	1:160	1:80	Yes (convalescent)	Yes
12	9	< 1:40	< 1:40	Yes (late acute)	n.d.
	45	< 1:40	1:160	Yes (convalescent)	n.d.
13	7	< 1:40	< 1:40	Yes (early acute)	n.d.
	16	1:40	1:80	Yes (convalescent)	n.d.

n.d., not done

### Serum cytokine, chemokine and growth factor measurements

**Fig. 2:**
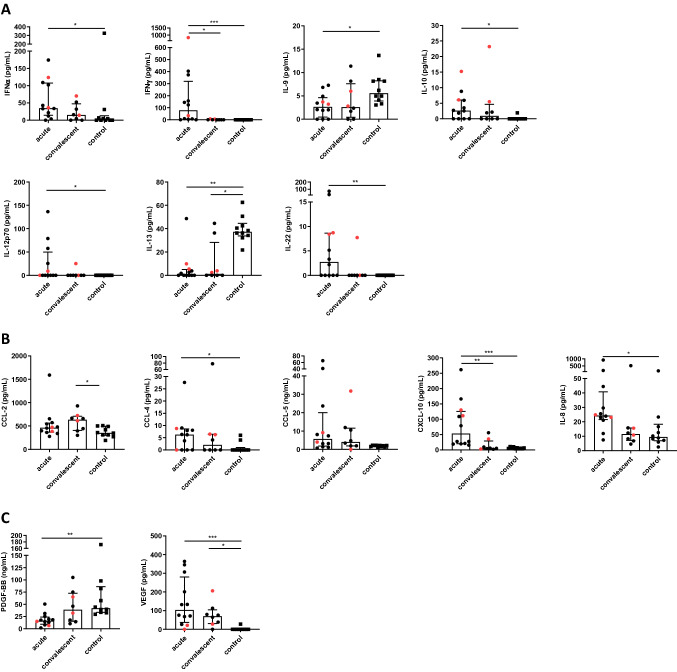
Cytokine, chemokine and growth factor levels in serum from patients with African tick bite fever and healthy controls. Serum cytokines (Panel **A**), chemokines (Panel **B**) and growth factors (Panel **C**) were analyzed from 2 hospitalized ATBF patients (red) and 11 ATBF patients without hospitalization (black) with bead-based LegendPlex assay (BioLegend, USA). Ten samples from healthy persons were analyzed in parallel. Illness was assigned to the acute phase for 13 samples (days 1–14) and to the convalescent phase for 8 samples (days 15–45). Data are expressed as median with interquartile range. Statistical analyses were performed using the Kruskal–Wallis test and subsequent Dunn’s multiple comparisons test. Asterisks indicate statistically significant differences: *p < 0.05, **p < 0.01, ***p < 0.001. CCL, CC-chemokine ligand; CXCL, C–X–C motif chemokine ligand; IFN, interferon; IL, interleukin; PDGF, platelet-derived growth factor; VEGF, vascular endothelial growth factor

Serum cytokine responses were analyzed by LegendPlex assay (BioLegend, USA) from all patients included in this study. Samples from eight patients were available at two different time points of infection (Table 2). For cytokine analysis, blood sampling dates from the patients were assigned to the acute phase of infection (days 1–14 of illness), and convalescent phase (days 15–45 of illness), respectively [3]. Illness was determined to be in the acute phase for 13 serum samples and in the convalescent phase for 8 samples (Fig. 2). Two serum samples from patient five were assigned to the acute phase of illness and the samples were measured separately but for the following analyzes the mean value was calculated (Fig. 2). In addition, the acute phase was further subdivided into an early acute phase (day 1–7, 7 samples) and a late acute phase (day 8–14, 6 samples) for a more detailed evaluation (Supplementary Fig. 1) as inspired by a report of Jensenius et al. [6]. Ten sera from healthy blood donors negative for antibodies against *R. africae* served as controls.

The detection limits of the LegendPlex assay for the analyzed cytokines were as follows:

CC-chemokine ligand 2 (CCL-2; N/A (not available)), CCL-3 (4.09 pg/mL), CCL-4 (3.68 pg/mL), CCL-5 (20.99 pg/mL), C-X-C motif chemokine ligand 10 (CXCL-10; N/A), fibroblast growth factor basic (FGFb; 18.57 pg/mL), eotaxin (N/A), granulocyte-colony stimulating factor (G-CSF; 20.90 pg/mL), granulocyte macrophage-colony stimulating factor (GM-CSF; 8.82 pg/mL), interferon α (IFNα; 3.11 pg/mL), IFNγ (2.41 pg/mL), interleukin 1ß (IL-1ß; 4.23 pg/mL), IL-2 (2.03 pg/mL), IL-4 (3.00 pg/mL), IL-5 (3.92 pg/mL), IL-6 (2.15 pg/mL), IL-8 (N/A), IL-9 (1.25 pg/mL), IL-10 (1.92 pg/mL), IL-12p70 (7.99 pg/mL), IL-13 (1.52 pg/mL), IL-17A (2.85 pg/mL), IL-17F (4.12 pg/mL), IL-21 (4.35 pg/mL), IL-22 (1.83 pg/mL), platelet-derived growth factor bb (PDGF-BB; N/A), tumor necrosis factor (TNF, 4.11 pg/mL) and vascular endothelial growth factor (VEGF; 19.14 pg/mL).

### Isolation and restimulation of peripheral blood mononuclear cells (PBMCs)

PBMCs were isolated from eight patients (Table 2), including sampling at two time points in three individuals, by density gradient centrifugation. For this purpose, ethylenediaminetetraacetic acid (EDTA) blood was diluted 1:3 with phosphate-buffered saline (PBS) and gently layered over Ficoll (Ficoll-Paque Plus, GE Healthcare Bio Sciences, Uppsala, Sweden). After centrifugation (600 g, 20 min), the intermediate layer was removed and washed with PBS. The pelleted cells were counted and cryopreserved in fetal calf serum (FCS; PAA Laboratories, Pasching, Austria)/10% dimethyl sulfoxide (DMSO) at − 80 °C overnight, followed by storage in liquid nitrogen until further use.

For the restimulation experiments, the cells were thawed and 37 °C warm RPMI 1640 medium supplemented with 2% HEPES buffer (PAN Biotech GmbH, Aidenbach, Germany), 5% FCS, 2 mM glutamine and 1% penicillin/streptomycin (Gibco, Carlsbad, USA) was added drop-wise to the cells. Cells were then centrifuged (430 g, 5 min). The washing step was repeated and the cells were incubated in medium for 3 h at 37 °C/5% CO_2_. After this resting period, the cells were washed again in medium and up to 10^6^ cells were incubated with medium containing 2.5 ng/mL phorbol-12-myristate-13-acetate (PMA; Sigma-Aldrich, Munich, Germany), 5 µg/mL ionomycin (Sigma-Aldrich, Steinheim, Germany) and 1 µg/mL Golgi-Plug (BD Pharmingen, Heidelberg, Germany) in a total volume of 200 µL for 4 h at 37 °C/5% CO_2_.

### Flow cytometry analyses of PBMCs

To be able to exclude dead cells from the analysis, PBMCs were incubated with the viability dye Zombie-NIR (1:1000 in PBS, Biolegend, San Diego, USA) for 30 min in the dark. After washing in PBS, cells were stained with antibodies against CD3 (clone UCHT1; 1:50) and CD8 (clone RPA-T8; 1:500; both from Biolegend, San Diego, USA) in PBS/2% bovine serum albumin (BSA) for 20 min at 4 °C in the dark. Then, cells were washed in PBS/2% BSA and permeabilized with Fixation and Permeabilization buffer (BD Pharmingen, Heidelberg, Germany) for 20 min at 4 °C in the dark. The cells were washed twice in Perm/Wash buffer (BD Pharmingen, Heidelberg, Germany) and stained intracellularly with antibodies against CD4 (clone RPA-T4; 1:20), IFNγ (clone 4S.B3; 1:100), IL-17 (clone BL168; 1:20), IL-22 (clone 2G12A41; 1:20) and TNF (clone MAb11; 1:100; all from Biolegend, San Diego, USA) in Perm/Wash buffer for 30 min at 4 °C in the dark. Afterwards, the cells were washed again in Perm/Wash buffer and resuspended in PBS. Analyses were performed with a BD LSR II flow cytometer (BD Biosciences, San José, USA) and FlowJo single cell analysis software (FlowJo LLC, Ashland, USA).

### Statistics

Statistical analysis was performed with GraphPad Prism 7 software (GraphPadSoftware Inc., La Jolla, USA). For comparison between the analyzed groups, the Kruskal–Wallis test with subsequent Dunn’s multiple comparisons test was used.

## Results

### Serum cytokine, chemokine and growth factor analyses

In total, the expression of 28 different cytokines, chemokines and growth factors was examined in the samples and significant differences in the serum concentrations of 14 of these factors were detected (Fig. 2, Supplementary Fig. 1).

In the first 2 weeks of illness, the expressions of the cytokines IFNα, IFNγ, IL-10, IL-12p70, and IL-22 were significantly increased compared to the healthy control group and then decreased again in the convalescent phase; this decrease was even significant for IFNγ. While the level of IFNγ was significantly increased in the early and late acute phase of the disease, IL-10 and IL-22 started to increase in the early acute phase of illness and were then significantly increased in the late acute phase in comparison to the control. Interestingly, the serum levels of IL-9 and IL-13 were lower than in the control samples at the time points investigated; however, it should be emphasized that the IL-13 concentrations in particular were significantly decreased at all time points examined and the IL-9 concentration was only significantly decreased in the acute phase of illness.

The concentrations of the chemokines CCL-4, CCL-5, CXCL-10 and IL-8 were significantly elevated in the acute phase of illness compared to the control group and then declined again in the convalescent phase; this decrease was even significant for CXCL-10. The further subdivision into the early and late acute phase showed that the concentrations of CCL-4, CCL-5 and CXCL-10 in the late acute phase were significantly higher than in the controls, whereas the concentration of IL-8 was similar in the early and late acute phase in sera from ATBF patients (Supplementary Fig. 1). In contrast to these four chemokines that were elevated in the acute phase of illness, the values of CCL-2 were significantly elevated in the convalescent phase compared to the control and comparable to the control group in the acute phase (Fig. 2).

The levels of two growth factors exhibited significant differences compared to the control. While the VEGF concentrations were significantly increased in all phases of the disease compared to the control, the levels of PDGF-BB were significantly reduced in the acute phase and especially in the early acute phase compared to the control.

Because of a more severe course of disease, two patients had to be hospitalized. The corresponding cytokine, chemokine and growth factor values were highlighted for comparison to the values of non-hospitalized ATBF patients. However, the values were scattered randomly so that no clear trend was visible.

No significant differences in the expression of CCL-3, eotaxin, FGFb, G-CSF, GM-CSF, IL-1ß, IL-2, IL-4, IL-5, IL-6 were detectable in the sera of the ATBF patients compared to controls, and this was also true for IL-17A, IL-17F, IL-21 and TNF—with the exception of one patient who showed elevated serum levels of these cytokines in the first week of illness (data not shown).

### T cell analyses

**Fig. 3:**
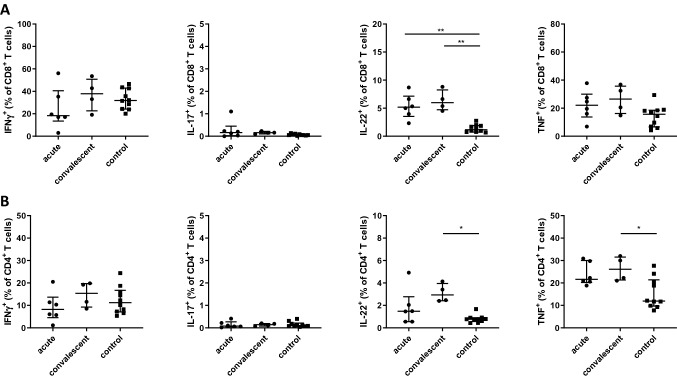
CD8^+^ and CD4^+^ T cell responses in patients with African tick bite fever and healthy controls. PBMCs were isolated and stimulated with PMA/Ionomycin and stained for CD3, and in addition for CD8 (Panel **A**) and CD4 (Panel **B**), as well as for IFNγ, IL-17A, IL-22 and TNF. Data are expressed as median with interquartile range. Statistical analyses were performed using the Kruskal–Wallis test and subsequent Dunn’s multiple comparisons test. Asterisks indicate statistically significant differences: *p < 0.05, **p < 0.01. IFN, interferon; IL, interleukin; TNF, tumor necrosis factor

As the effector cytokines IFNγ and TNF seem to be essential in combating rickettsial infections, and a protective function is also assigned to IL-17 or IL-22 [9], such T cell responses at different time points of illness were also analyzed in the patient cohort. These investigations showed that the percentage of IFNγ-producing CD3^+^ T cells as well as the CD4^+^ T cell population and the CD8^+^ T cells were comparable between ATBF patients and controls. The situation was different for the cytokine TNF, while the percentage of TNF producing CD8^+^ T cells was comparable between patients and controls, the percentage of TNF expressing CD4^+^ T cells was generally increased in the patients at all time points examined (Fig. 3). This percentage was even significantly elevated in the convalescent phase.

Interestingly, both CD4^+^ and CD8^+^ T cells from the patients produced IL-22 (Fig. 3). The percentage of IL-22 producing CD8^+^ T cells from ATBF patients was generally higher than those from the controls, and was significantly elevated in the acute phase of illness and in the convalescent phase. A significant increase in IL-22 expressing CD4^+^ T cells was also detected in the convalescent phase compared to the control group, but not in the acute phase of ATBF. This IL-22 production, however, did not originate from T helper (Th) 17 cells which can secrete IL-22 as well as IL-17, since no co-expression of these cytokines was detectable in CD4^+^ T cells of the patients; this is, of note, also true for the CD8^+^ T cell population. In general, only very few IL-17 producing T cells could be detected in both the CD4^+^ T cell population and in the CD8^+^ T cell population and the percentage did not differ from those in the control group. Co-expression of IFNγ was detected in IL-22 producing CD4^+^ and CD8^+^ T cells of the patients in the different phases of the disease (median for CD4^+^ T cells: d1–14: 33%, d15–39: 27%; median for CD8^+^ T cells: d1–14: 51%, d15–39: 42%).

## Discussion

Little is known about immune responses during rickettsial infections in humans. In this study, we analyzed serum concentrations of different chemokines, cytokines, growth factors and T cell responses in 13 ATBF patients.

Serum levels of the chemokines CCL-4 (also known as MIP-1ß), CCL-5 (also known as RANTES), CXCL-10 (also known as IP-10), and IL-8 increased in the acute phase of illness and then decreased again in the convalescent phase, although they did not reach the level of the control at the last time point examined. While IL-8 was detectable at elevated levels in the first 2 weeks of illness, the concentrations of CCL-4, CCL-5 and CXCL-10 started to increase in the first week of illness and peaked in the second week. An enhanced expression of CCL-4, CXCL-10 and IL-8 in the acute phase of illness was also observed in sera from patients infected with TG rickettsiae [12]. Likewise, the chemokine concentrations started to increase in the first week of illness and reached their peak in the second week of illness. Thus, in infections with different rickettsial species, some similar immune mechanisms appear to take place in a similar chronological order. While no increased expression of CCL-5 was detected in TG rickettsiosis, the expression of CCL-5 and also CXCL-10 was elevated in patients with Mediterranean spotted fever (MSF) caused by *R. conorii* and RMSF caused by *R. rickettsii* [13, 14].

In contrast to the above-mentioned chemokines that were elevated in the acute phase of ATBF, the concentrations of CCL-2 (also known as MCP-1) were significantly elevated in the convalescent phase compared to the control and similar to the control group in the acute phase (Fig. 2). Of these chemokines, CCL-2 has both proinflammatory and anti-inflammatory properties, since both antigen-presenting cells and T cells express the associated receptor, as do regulatory T cells [15]. It is, therefore, conceivable that CCL-4, CCL-5, CXCL-10 and IL-8 would initiate and maintain the inflammatory response in the acute phase of infection and the increased release of CCL-2 during the convalescent phase would regulate the immune response in order to reduce immune pathologies.

While CCL-2, CCL-4, CCL-5 and IL-8 each act chemotactic on several different cell populations including T cells, CXCL-10 solely has chemotactic effects on T cells [16]. Interestingly, the expression of CXCL-10 and also CXCL-9, both T cell attracting chemokines that bind to the same chemokine receptor CXCR-3, peaked a few days before T cells infiltrated the infected tissues of *R. conorii* infected mice [14]. T cells play a crucial role in pathogen defense and both CD4^+^ and CD8^+^ T cells and their immune mediators have been shown to be protective in rickettsial infections [17–20]. Especially the release of their effector cytokines IFNγ and TNF and the cytotoxic activity of CD8^+^ T cells seem to be important for the elimination of rickettsia (reviewed in [9]). The serum concentrations of IFNγ in ATBF patients of this study were significantly increased compared to controls in the first 2 weeks of illness and then decreased again. However, the increased IFNγ levels did not seem to originate from T cells as the percentage of IFNγ producing CD3^+^ T cells as well as the subpopulations CD4^+^ and CD8^+^ T cells were similar between patients and controls. In addition to T cells, natural killer cells [21, 22], and professional antigen-presenting cells like B cells [23–26], dendritic cells and macrophages [27, 28] can produce IFNγ and thus contribute to the defense against rickettsiae. In *R. conorii*-infected mice, for example, NK cells appear to contribute to the early immune response against rickettsiae by a mechanism involving IFNγ [29]. Furthermore, several other studies of murine rickettsial infections indicate an important role of NK cell derived IFNγ in the early defense of the pathogens [9]. Whether NK cells also play a role in the early immune response against rickettsiae in humans and whether the increased release of IFNγ originates from NK cells is unclear and needs to be further investigated.

The percentage of TNF expressing CD4^+^ T cells was increased in the patients at all time points examined while CD8^+^ T cells from patients did not produce more of this cytokine than those from healthy controls. Furthermore, the concentrations of TNF in sera were similar in patients and controls—with the exception of one patient who showed elevated serum levels of this cytokine in the first week of illness. Unfortunately, no T cell responses could be examined in this patient. TNF and IFNγ synergistically lead to the expression of the inducible nitric oxide synthase in macrophages [30–32] and thus can contribute to the elimination of rickettsiae.

The IL-22 concentration in the sera of ATBF patients was significantly elevated in the acute phase of illness and later declined. IL-22 appears to play a protective role in microbial defense and this cytokine is involved in tissue regeneration and damage protection [33]. It induces the expression of tissue-specific proteins that are involved in tissue inflammation, immunosurveillance and homeostasis [34–37]. In a mouse model of TG rickettsiosis, IL-22 seems to support bacterial elimination and thus has protective functions [17]. In addition, it also seems to play a role in infections with TG rickettsiosis in humans, since its expression is upregulated in these infections in the acute phase and also on days 15 to 28 of illness [12]. IL-22 is produced by various types of innate lymphoid cells and also by cells of the adaptive immune system like activated T cells. Today, it is known that in humans, Th1, Th17, and Th22 cells are the major CD4^+^ T cell subsets producing IL-22 but CD8^+^ T cells can also secrete IL-22 [38]. In this study, both CD4^+^ and CD8^+^ T cells of ATBF patients produced IL-22 and the percentage of IL-22 producing CD8^+^ T cells was even higher than those of the CD4^+^ T cell subpopulation. Whether IL-22 was mainly produced by antigen-specific T cells or by bystander T cells needs to be further investigated.

IL-22 was partly produced by T cells that co-expressed IFNγ and IL-22 but not by T cells co-producing IL-22 and IL-17. This is in contrast to the TGR mouse model where Th17 cells were found to be the source of protective IL-22 [17]. Thus, in ATBF both beneficial cytokines IFNγ and IL-22 could work in concert. Whether IL-22 has a protective effect in the human host and what function it assumes during rickettsial infections remains to be investigated. However, it does not appear to be used for communication between immune cells, since these cells do not express the complete IL-22 receptor [36, 39–41]. Rather, it has been shown that tissue cells such as epithelial cells and fibroblasts or keratinocytes and hepatocytes can react to IL-22 and that the responsiveness of cells to IL-22 can be elevated under inflammatory conditions by cytokines like IFNγ or TNF [33, 38, 40, 42, 43]. Antimicrobial mechanisms and repair mechanisms could thus be strengthened.

While the serum levels of IL-13 were significantly decreased, the anti-inflammatory cytokine IL-10 was increased in ATBF patients compared to controls and during all time points examined in this study. Decreased IL-13 concentrations in the sera of ATBF patients were also described in a previous study in which the concentrations of IL-13 tended to increase again during follow-up [44]. IL-10 contributes to the regulation of immune responses by inhibiting the production of proinflammatory cytokines and thus can help to minimize immunopathology during rickettsial infections.

The concentration of the growth factor VEGF was significantly increased in ATBF patients at all times examined in this study. It is one of the most important survival and growth factors of the vascular endothelium. VEGF receptors are mainly expressed on endothelial cells, and VEGF appears to be important for the induction of endothelial cell proliferation [45]. Therefore, an increased release of VEGF could contribute to the strengthening and repair of the endothelium during rickettsial infections.

In summary, the present study has shown the release of proinflammatory as well as anti-inflammatory immune mediators during the course of molecularly confirmed ATBF at different time points of illness. Especially the expression of the T cell attracting chemokine CXCL-10 was increased at all time points examined, and in the first 2 weeks of illness also the proinflammatory cytokine IFNγ that seem to be a key cytokine for the elimination of rickettsiae. In contrast to TNF, which was secreted by CD4^+^ T cells, IFNγ was not increasingly derived from T cells. Future studies should further investigate which immune cell populations are responsible for the secretion of IFNγ during rickettsial infections. CD4^+^ T cells as well as CD8^+^ T cells were responsible, at least in part, for the increased IL-22 production in the first 2 weeks of illness in ATBF patients. The role of IL-22 during rickettsial infections is not yet entirely clear and needs to be further investigated, e.g., what effect IL-22 has on responsive cells during infection. As a limitation of the study, we have included only 13 patients. However, these cases were unequivocally confirmed ATBF cases by molecular detection and sequencing of the causative pathogen, and comprise a well-characterized patient group. Moreover, fever was not recorded in all patients as a hallmark symptom of a clinically obvious systemic inflammatory response, and as thus, cytokine and activation levels in such patients could be low. However, other symptoms of systemic involvement such as fatigue, apathy, or headache were also present in some patients, and except for two patients, at least one of these symptoms was recorded. Moreover, only two patients had a severe course of the disease and had to be hospitalized. It is, therefore, difficult to compare the cytokine profiles according to the severity of the disease. In our study, serum instead of plasma was used. During coagulation, this could theoretically lead to lower levels of cytokines and chemokines.

Despite these limitations, the results of this study broaden the knowledge of immune responses to *R. africae* infections during successive weeks of illness. However, further studies for the identification of protective immune responses including T cell responses in rickettsiosis patients are needed.

## Supplementary Information

Below is the link to the electronic supplementary material.

Supplementary Figure 1 Cytokine, chemokine and growth factor levels in serum from patients with African tick bite fever in the early acute, late acute and convalescent phase, and healthy controls. Serum cytokines (Panel A), chemokines (Panel B) and growth factors (Panel C) were analyzed from 2 hospitalized ATBF patients (red) and 11 ATBF patients without hospitalization (black) with bead-based LegendPlex assay (BioLegend, USA). Ten samples from healthy persons were analyzed in parallel. Illness was assigned to the early acute phase for 7 samples (days 1-7), the late acute phase for 6 samples (days 8-14) and to the convalescent phase for 8 samples (days 15-45). Data are expressed as median with interquartile range. Statistical analyses were performed by using the Kruskal-Wallis test and subsequent Dunn`s multiple comparisons test. Asterisks indicate statistically significant differences: *p<0.05, **p<0.01. CCL, CC chemokine ligand; CXCL, C-X-C motif chemokine ligand; IFN, interferon; IL, interleukin; PDGF, platelet derived growth factor; VEGF, vascular endothelial growth factor (PPTX 151 kb)

## Data Availability

The datasets generated and analyzed during the current study are available from the corresponding author on reasonable request.
