# Supplementary figures and images for: Inflammatory cytokine profile and T cell responses in African tick bite fever patients
Source: Med Microbiol Immunol. 2022 May 11;211(2-3):143–52. doi: 10.1007/s00430-022-00738-5 (PMC9092931; doi:10.1007/s00430-022-00738-5)

## Slide 1
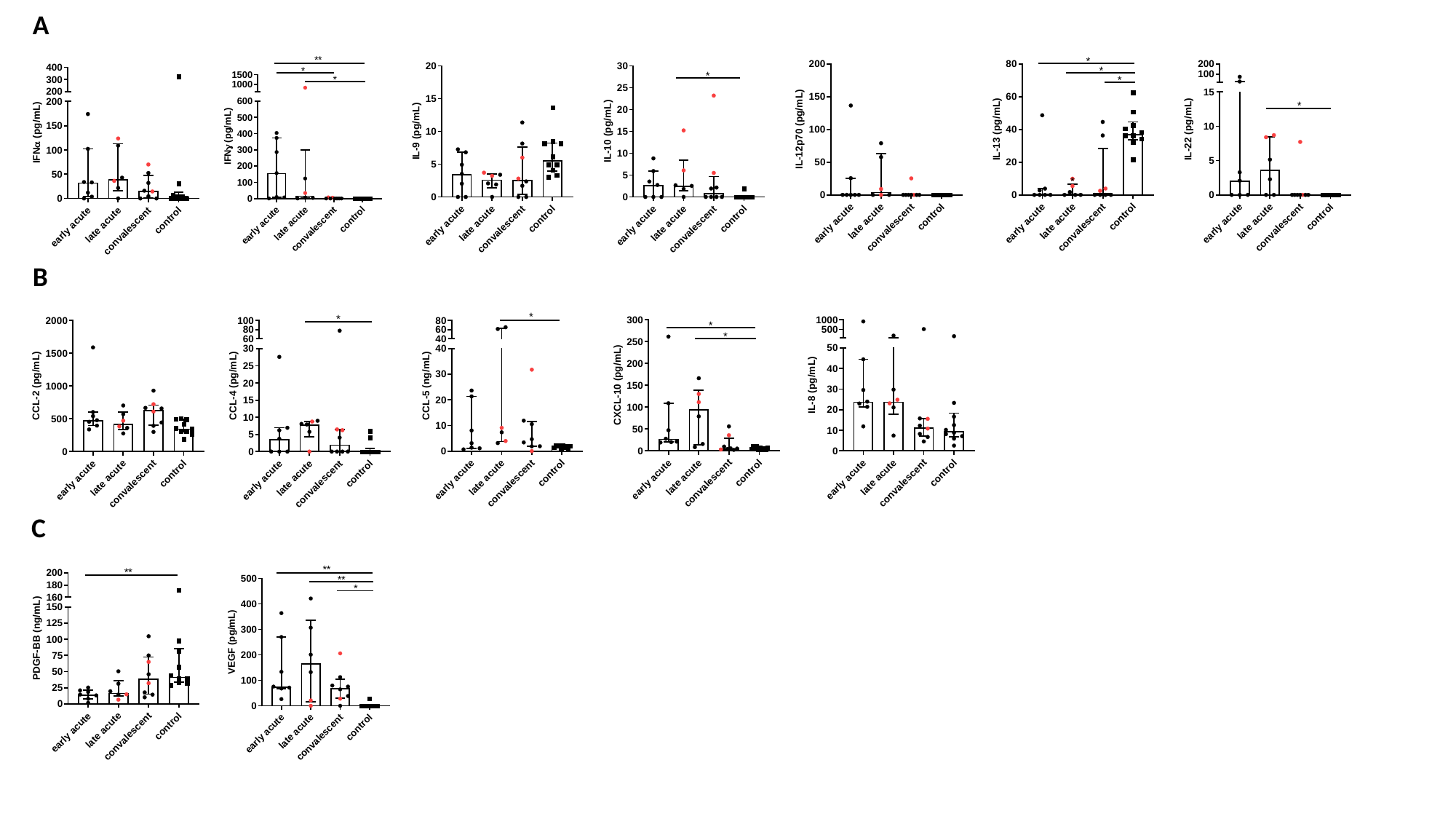

A
B
C

Supplement: Supplementary file 1 — Supplementary Figure 1 Cytokine, chemokine and growth factor levels in serum from patients with African tick bite fever in the early acute, late acute and convalescent phase, and healthy controls. Serum cytokines (Panel A), chemokines (Panel B) and growth factors (Panel C) were analyzed from 2 hospitalized ATBF patients (red) and 11 ATBF patients without hospitalization (black) with bead-based LegendPlex assay (BioLegend, USA). Ten samples from healthy persons were analyzed in parallel. Illness was assigned to the early acute phase for 7 samples (days 1-7), the late acute phase for 6 samples (days 8-14) and to the convalescent phase for 8 samples (days 15-45). Data are expressed as median with interquartile range. Statistical analyses were performed by using the Kruskal-Wallis test and subsequent Dunn`s multiple comparisons test. Asterisks indicate statistically significant differences: *p<0.05, **p<0.01. CCL, CC chemokine ligand; CXCL, C-X-C motif chemokine ligand; IFN, interferon; IL, interleukin; PDGF, platelet derived growth factor; VEGF, vascular endothelial growth factor (PPTX 151 kb) [file 430_2022_738_MOESM1_ESM.pptx]
